# COMPASSIONATE CAREGIVING: CREATING A CNA LEADERSHIP ACADEMY

**DOI:** 10.1093/geroni/igae098.0898

**Published:** 2024-12-31

**Authors:** Monica Long, Lauren Gleason, Jeffrey Graupner, Kanika Mittal, Katherine Thompson

**Affiliations:** University of Chicago Medicine/SHARE Network, Glenwood, Illinois, United States; University of Chicago Medicine, Chicago, Illinois, United States; University of Chicago, Chicago, Illinois, United States; University of Chicago Medicine, Chicago, Illinois, United States; University of Chicago, Chicago, Illinois, United States

## Abstract

Certified Nursing Assistants (CNAs) provide direct patient care such as bathing, dressing, feeding, as well as reporting changes in patients’ conditions under the supervision of licensed nurses. Despite the crucial role CNAs play, this role is characterized by high turnover rates and relatively low wages compared to other healthcare roles. SHARE Network at UChicago Medicine created a CNA Leadership Academy with an aim to retain and empower CNAs to cultivate their potential as leaders in their respective healthcare facilities. This initiative comprised of a comprehensive curriculum design to enhance CNAs understanding of leadership principles, communication techniques, quality improvement (QI), resilience and self-care techniques using Project ECHO, an educational model that provides tele-mentoring to healthcare workers. Kern’s 6-step approach for curriculum development, mixed-methods pre/post survey, qualitative analysis for curriculum evaluation and a 7-point Likert Scale was used for evaluation. Participants in 2 cohorts were mostly African Americans and resulted Likert scores of, 5=Competent, 6=Very competent, 7=Expert post training. Participants created QI projects utilizing Stop & Watch communication tools, fostering a culture of communication among healthcare professionals, resilience, self-care, and a team approach to patient care. Within the CNA Leadership Academy, CNAs are better equipped to advocate for patients, address emerging challenges, and drive positive change within their healthcare organizations. The CNA Leadership Academy educational initiative represents a proactive approach to nurturing leadership talent within the CNA workforce. Equipping CNAs with knowledge and support, the program initiative contributes to resilience and effective healthcare, ultimately improving outcomes for patients and healthcare providers alike.

